# Clinical presentations, treatments, and outcomes of pediatric lupus nephritis: a prospective cohort study from the Pediatric Nephrology Research Consortium

**DOI:** 10.1007/s00467-026-07329-z

**Published:** 2026-05-11

**Authors:** Melonie Phillips, Mahmoud Kallash, Chloe Tang, Steve Rust, Kia Jubert-Bacon, Scott Wenderfer, Smriti Mohan, Neal Blatt, Don Batisky, Alejandro Quiroga, Tetyana L. Vasylyeva, Nilka DeJesus, Jerome C. Lane

**Affiliations:** 1 The Division of Nephrology and Hypertension, Nationwide Children’s Hospital, Columbus, OH USA; 2 The Research Institute, Nationwide Children’s Hospital, Columbus, OH USA; 3 The Division of Pediatric Nephrology, Texas Children’s Hospital, Houston, TX USA; 4 The Division of Pediatric Nephrology, CS Mott Children’s Hospital, MI, Ann Arbor, USA; 5 The Division of Nephrology, Emory University Hospital, Atlanta, USA; 6 The Division of Pediatric Nephrology, Helen DeVos Children’s Hospital, MI Grand Rapids, USA; 7 The Division of Nephrology, Texas Tech University Health Sciences Center, Lubbock, TX USA; 8 The Pediatric Nephrology Section, University of Puerto Rico, San Juan, Puerto Rico; 9 The Division of Pediatric Nephrology, Ann & Robert H. Lurie Children’s Hospital of Chicago, Chicago, IL USA; 10 The University of British Columbia, Vancouver, BC Canada; 11 Corewell Health William Beaumont University Hospital, Royal Oak, MI USA; 12 Cincinnati Children’s Hospital Medical Center, Cincinnati, OH USA; 13 Children’s Mercy Kansas City, Kansas City, MO USA

**Keywords:** Pediatric lupus nephritis, Pediatric systemic lupus erythematosus, Pediatric glomerulonephritis

## Abstract

**Background:**

There are few prospective studies of mycophenolate mofetil (MMF) versus cyclophosphamide (CYC) for pediatric lupus nephritis (pLN) and none evaluating rituximab (RTX).

**Methods:**

The Prospective Pediatric Lupus Nephritis Registry (ProPeL-R) enrolled patients < 21 years within 4 weeks of an initial kidney biopsy diagnostic of pLN. Demographic, clinical, and laboratory data were collected prospectively at enrollment, 3 months, 6 months, and then every 6 months thereafter for up to 5 years of follow-up. For this study, we compared patients receiving initial therapy with corticosteroids (CS) and either MMF (*n* = 33) vs. CYC (*n* = 18), and those treated with CS, either MMF or CYC, with RTX (*n* = 20) vs. without RTX (*n* = 51).

**Results:**

Histology consisted of 18% class III; 35% class IV; 25% mixed class; and 22% pure class V. Eighty-two percent were female. No significant differences in response or infection rates were identified between those receiving MMF or CYC. There was a significant increase in combined complete (CR) and partial response (PR) at 24 months with RTX use. Overall CR rates at 6 and 24 months were 35% and 58%, respectively. At 24 months, 51% of patients continued on CS, and 56% of patients experienced at least one CS side effect.

**Conclusions:**

MMF and CYC had similar efficacy as initial therapies for pLN. RTX may augment long-term response. However, response rates were suboptimal. Large variations in initial therapy were observed. Given the paucity of prospective data in pLN, our study further illustrates the need for data-driven, pediatric-specific protocols to standardize care and improve outcomes.

**Graphical Abstract:**

**Figure:**
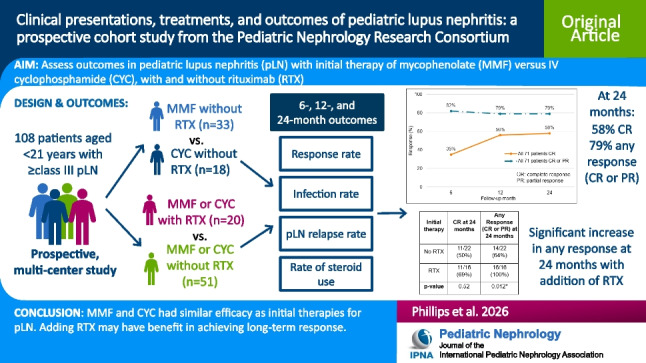
A higher resolution version of the Graphical abstract is available as Supplementary information.

**Supplementary Information:**

The online version contains supplementary material available at 10.1007/s00467-026-07329-z.

## Introduction

Systemic lupus erythematosus (SLE) is an autoimmune disease that can affect multiple organ systems, including the kidneys (lupus nephritis, LN) [1]. In childhood-onset SLE (cSLE), kidney involvement (pLN) usually occurs within 5 years of cSLE diagnosis but may be the first manifestation of cSLE [1]. Children with pLN tend to have more severe disease and worse long-term outcomes compared to adults for unclear reasons [2, 3]. The Kidney Disease Improving Global Outcomes (KDIGO) LN guideline recommends tailoring immunosuppressive treatment to LN biopsy classification [4]. The American College of Rheumatology (ACR) 2024 LN guidelines include pediatric-specific Good Practice Statements, but these were based on observational trials, extrapolation from adult LN study data, or pediatric rheumatology and nephrology expert opinion [5]. Differences in pediatric psychosocial development and physiology may not be suitable for adult-based management. Additionally, treatment options for pLN are limited as many therapies are inaccessible without regulatory approval.

To our knowledge, only three prospective, multicenter pLN studies exist [6, 7] with only one that enrolled patients from the United States (US) [8]. None have evaluated rituximab (RTX) as an adjunctive agent for initial therapy. Several international registries that compile clinical information about patients with SLE and LN exist. Examples include the United Kingdom Juvenile-onset Systemic Lupus Erythematosus (UK JSLE) Cohort Study and Repository and the LN Registry jointly founded by the European Society for Pediatric Nephrology (ESPN) and the European Rare Kidney Disease Reference Network (ERKNet).

Given the need for prospective real-world data regarding pLN treatment and outcomes for patients in the US, the Prospective Pediatric Lupus Nephritis Registry (ProPeL-R) was created through the Pediatric Nephrology Research Consortium (PNRC). Patients with cSLE < 21 years of age were enrolled in ProPeL-R within 4 weeks of an initial kidney biopsy diagnostic for pLN, with follow-up for up to 5 years. Using ProPeL-R data, we aimed to characterize the clinical presentations and initial regimens used in pLN; compare the efficacy of mycophenolate (MMF) and IV cyclophosphamide (CYC) for initial therapy; assess the benefit of RTX as an adjunctive agent for initial therapy; and evaluate secondary outcomes, including corticosteroid (CS) therapy duration and side effects, types and rates of infection, and rates of pLN relapse.

## Materials and methods

### Patients and data collection

The ProPeL-R enrolled 129 patients with biopsy-proven class II–VI pLN from eight centers between 2011 and 2019. Participating centers were Ann & Robert H. Lurie Children’s Hospital of Chicago (Chicago, IL); Nationwide Children’s Hospital (Columbus, OH); Texas Children’s Hospital (Houston, TX); Texas Tech University Health Sciences Center (Lubbock, TX); CS Mott Children’s Hospital (Ann Arbor, MI); Helen DeVos Children’s Hospital (Grand Rapids, MI); Emory University Hospital (Atlanta, GA); and University of Puerto Rico Hospital (San Juan, Puerto Rico). Demographic, clinical, and laboratory data were collected prospectively starting within four weeks of initial kidney biopsy, at 3- and 6-month follow-ups, and then every six months thereafter for up to five years of follow-up. Initial therapy was defined as treatment received within the initial 6 months after diagnosis and maintenance therapy as treatment started thereafter. Treatment was according to each center’s practice.

For this manuscript, only patients with pLN class III, IV, pure V, or mixed class (either class III + V or class IV + V), based on the International Society of Nephrology/Royal Pathology Society (ISN/RPS) [9], who had recorded data beyond the first visit were included, totaling 108 patients. Biopsies were interpreted by the local center’s pathologists and did not go through central review. For these patients, we evaluated demographics, plasma and urine labs, and the presence of acute kidney injury (AKI) and hypertension (HTN) at the time of diagnosis; initial therapy used; and the need for chronic dialysis or kidney transplant during the five years of follow-up.

**Fig. 1:**
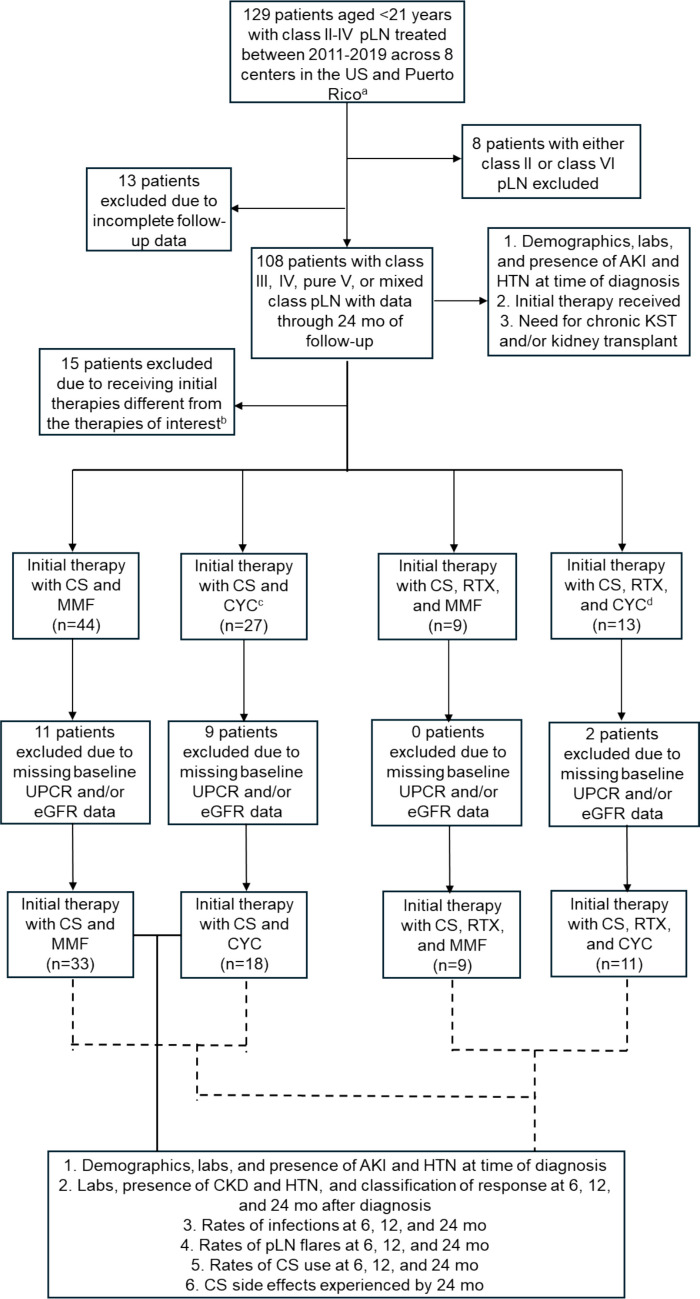
Flowchart of patient selection. Corticosteroids (CS) include both IV and enteral formulations. Mycophenolate (MMF) includes both mycophenolate mofetil and mycophenolic acid. IV cyclophosphamide (CYC) encompasses both EuroLupus and the National Institutes of Health (NIH) dosing protocols. Rituximab (RTX) encompasses both weekly and bi-monthly dosing protocols. The different subset analyses are illustrated at the bottom of the flowchart with a solid line (MMF vs. CYC) and a dashed line (RTX vs. no RTX). Abbreviations: AKI, acute kidney injury; CKD, chronic kidney disease; CS, corticosteroid; CYC, IV Cyclophosphamide; eGFR, estimated glomerular function rate; HTN, hypertension; KST, kidney support therapy; pLN, pediatric lupus nephritis; MMF, mycophenolate; mo, months; RTX, rituximab; UPCR, urine protein-to-creatinine ratio; US, United States. ^a^Participating centers were Ann & Robert H. Lurie Children’s Hospital of Chicago (Chicago, IL); Nationwide Children’s Hospital (Columbus, OH); Texas Children’s Hospital (Houston, TX); Texas Tech University Health Sciences Center (Lubbock, TX); CS Mott Children’s Hospital (Ann Arbor, MI); Helen DeVos Children’s Hospital (Grand Rapids, MI); Emory University Hospital (Atlanta, GA), and University of Puerto Rico Hospital (San Juan, Puerto Rico). ^b^For the 15 patients with initial treatments not included in this study, they received the following: CS, MMF, and CYC (*n* = 5); CS (*n* = 3); CS, MMF, and tacrolimus (*n* = 2); CS, MMF, CYC, and RTX (*n* = 2); CS, MMF, and azathioprine (*n* = 1); CS, MMF, and Benlysta (*n* = 1); and MMF (*n* = 1). ^c^The dosing protocols for the 27 patients that received CYC were as follows: NIH protocol (*n* = 26) and EuroLupus protocol (*n* = 1). ^d^The dosing protocols for the 13 patients that received CYC and RTX were as follows: NIH protocol (*n* = 10) and EuroLupus protocol (*n* = 3)

Sub-analyses based on initial therapy were then performed at 6-, 12-, and 24-month follow-ups to evaluate differences in plasma and urine labs; prevalence of chronic kidney disease (CKD) and HTN; response rates; and rates of CS use, infections, and pLN relapses. Outcomes through 24 months were evaluated as this time period included more complete data. Initial regimens of interest included CS and MMF (either mycophenolate mofetil or mycophenolic acid) with or without RTX and CS and CYC (either standard NIH or EuroLupus protocols) with or without RTX. Of the 108 patients, a total of 37 patients were excluded, 15 due to receiving an initial regimen differing from the ones of interest and 22 due to incomplete documentation of urine protein-to-creatinine ratio (UPCR) and/or creatinine at time of diagnosis. Therefore, a total of 71 patients were included in these sub-analyses. Figure 1 summarizes patient selection and data of interest for this study.

The presence and stage of AKI at time of diagnosis were evaluated by calculating an estimated normal creatinine using methodology described by Hessey et al*.* [10] and comparing this estimate to the lab-measured creatinine. Definitions of AKI staging used are from the KDIGO 2012 AKI guidelines [11]. CKD staging at 6-, 12-, and 24-month follow-up was performed by calculating an estimated glomerular filtration rate (eGFR) using the “Chronic Kidney Disease in Children under 25” (CKiD U25) equation [12, 13] and assigning the appropriate stage using eGFR definitions from the KDIGO 2024 CKD guidelines [14].

Proteinuria was recorded as UPCR. Discrete values for UPCR were used when determining type of response, but for presenting this data, a patient’s UPCR was categorized as normal (UPCR ≤ 0.2 mg/mg), sub-nephrotic range (UPCR > 0.2–≤2 mg/mg), and nephrotic-range (UPCR > 2 mg/mg). Hematuria was defined as ≥ 5 red blood cells per high-powered field (hpf) [15]. Pyuria was defined as ≥ 5 white blood cells per hpf [16].

Blood pressure was evaluated according to percentiles for a patient’s height and sex. HTN was defined as either (1) systolic blood pressure (SBP) or diastolic blood pressure (DBP) ≥ 95th percentile for patients aged < 13 years or (2) SBP ≥ 130 or DBP ≥ 80 for patients aged ≥ 13 years [17].

### Definitions of kidney outcomes

The criteria for pLN treatment response definitions included below were adapted from the 2012 Consensus Treatment Plans by the Childhood Arthritis and Rheumatology Research Alliance [18]. Proteinuria criteria were based on those used by De Mutiis et al*.* [3], which define the absence of abnormal proteinuria as UPCR ≤ 0.2 mg/mg. Criteria for changes in eGFR were based on the 2024 Lupus Nephritis KDIGO guidelines [4]. As with current clinical trial LN response definitions, hematuria was not included in response definitions [4].

A complete response (CR) was defined as UPCR ≤ 0.2 mg/mg and kidney function stabilization or improvement with either eGFR ≥ 90 mL/min/1.73 m^2^ or an eGFR decrease ≤ 15% compared to the eGFR measurement at time of diagnosis (defined as baseline eGFR).

A partial response (PR) was defined as a UPCR decrease of ≥ 50% from the time of diagnosis (defined as baseline UPCR), and if previously nephrotic, ≤ 2 mg/mg, as well as kidney function stabilization or improvement with either eGFR ≥ 90 mL/min/1.73 m^2^ or eGFR decrease of ≤ 15% from baseline.

No response was defined as a UPCR decrease of <50% from the time of diagnosis or a UPCR >2 mg/mg, as well as an eGFR decrease of >15% from baseline.

For subsequent analyses, CR and any response (denoted as CR + PR) were evaluated in order to assess characteristics for the best outcome, CR, and for any responsiveness to treatment.

pLN relapses were defined according to each center’s individual criteria.

### Data collection and statistical analyses

Study data were collected and managed using REDCap electronic data capture tools hosted at Northwestern University and the Northwestern University Clinical & Translational Sciences Institute (NUCATS). Research Electronic Data Capture (REDCap) is a secure, web-based software platform designed to support data capture for research studies, providing (1) an intuitive interface for validated data capture; (2) audit trails for tracking data manipulation and export procedures; (3) automated export procedures for seamless data downloads to common statistical packages; and (4) procedures for data integration and interoperability with external sources [19, 20]. NUCATS is funded in part by a Clinical and Translational Science Award (CTSA) grant from the National Institutes of Health (NIH) UL1TR001422.

Demographic variables and patient characteristics were characterized by median and interquartile range for continuous variables and by percentages for categorical variables. Tests of association were performed for biopsy class with laboratory measurements and presence of AKI and HTN at time of diagnosis. Fisher’s exact tests were performed for categorical variables; analysis of variance procedures were performed for continuous variables with no significant departures from normality; and Kruskal–Wallis procedures were performed for continuous variables with significant departures from normality. When significant differences across biopsy classes were found, pairwise comparisons were performed by Fisher’s exact test, *t*-test, or Mann–Whitney test, respectively.

For patients who received MMF or CYC, both with and without RTX, for initial therapy, tests of association were performed for initial therapy with laboratory measurements, presence of AKI (at diagnosis only), presence of CKD (at follow-up only), and presence of HTN at time of diagnosis and at follow-up. Fisher’s exact tests were performed for categorical variables; *t*-tests were performed for continuous variables with no significant departures from normality; and Mann–Whitney tests were performed for continuous variables with significant departures from normality. Tests of association, employing Fisher’s exact test procedure, were performed for response rates by biopsy class and initial therapy and rates of infection, pLN relapses, and CS use at 6-, 12-, and 24-month follow-ups.

## Results

###  Composite cohort—demographics, characteristics, laboratory data, and initial regimens (108 patients)

The median age of pLN onset was 14 years, and the oldest patient at the time of pLN onset was 19 years. The majority of affected patients were female (82%). Most patients self-identified as African American (35%), Hispanic (21%), or Caucasian (20%). The most common biopsy class was class IV (35%). There were no significant demographic differences between initial therapy subsets, except that class IV more often received CYC (*p* = 0.0024) (Table 1).

**Table 1:** Demographics and patient characteristics for the 108-patient cohort (labeled composite) and each subset based on initial therapy received, including the 33 patients who received corticosteroids (CS) and mycophenolate (MMF) (labeled MMF); the 18 patients who received CS and IV cyclophosphamide (CYC) (labeled CYC); the 51 patients who received CS and either MMF or CYC without Rituximab (RTX) (labeled No RTX); and the 20 patients who received CS and either MMF or CYC with RTX (labeled RTX). For categorical values, number of affected patients and the corresponding percentages are provided. For numerical data, median values and their corresponding lower quartiles (Q1) and upper quartiles (Q3) are provided

	Composite	MMF	CYC	***p***-value^a^	No RTX	RTX	***p***-value^b^
**Number of patients**	108	33	18		51	20	
**Median age in years at SLE diagnosis (Q1, Q3)**	14 (12, 16)	14.5 (12, 16)	13 (11, 16)		14 (11, 16)	14 (11, 16)	
**Median age in years at pLN diagnosis (Q1, Q3)**	14 (12, 16)	15 (13,16)	14 (12, 16)	0.64	15 (12, 16)	14 (11, 17)	0.81
**Sex assigned at birth**				1.00			0.45
Female	89 (82%)	29 (88%)	16 (89%)		45 (88%)	16 (80%)	
Male	19 (18%)	4 (12%)	2 (11%)		6 (12%)	4 (20%)	
**Self-identified race**				0.74			0.30
African American	38 (35%)	12 (36%)	5 (28%)		17 (33%)	6 (30%)	
Asian^c^	7 (6%)	3 (9%)	3 (17%)		6 (12%)	0 (0%)	
Caucasian	22 (20%)	9 (27%)	3 (17%)		12 (24%)	3 (15%)	
Hispanic	23 (21%)	5 (15%)	4 (22%)		9 (18%)	7 (35%)	
Other^d^	18 (18%)	4 (12%)	3 (17%)		7 (14%)	4 (20%)	
**Insurance type**				0.41			0.18
Medicaid	51 (47%)	16 (49%)	6 (33%)		22 (43%)	13 (65%)	
Private	48 (44%)	12 (36%)	11 (61%)		23 (45%)	6 (30%)	
Self-pay	3 (3%)	1 (3%)	0 (0%)		1 (2%)	1 (5%)	
Not reported	6 (6%)	4 (12%)	1 (6%)		5 (10%)	0 (0%)	
**Biopsy class**				0.0024*			0.43
Class III	19 (18%)	7 (21%)	2 (11%)		9 (18%)	7 (35%)	
Class IV	38 (35%)	8 (24%)	12 (67%)		20 (39%)	5 (25%)	
Mixed class^e^	27 (25%)	6 (18%)	4 (22%)		10 (20%)	4 (20%)	
Pure class V	24 (22%)	12 (36%)	0 (0%)		12 (23%)	4 (20%)	

Abbreviations: *CYC*, IV cyclophosphamide; *MMF*, mycophenolate; *pLN*, pediatric lupus nephritis; *RTX*, Rituximab; *SLE*, systemic lupus erythematosus

^a^These *p*-values are for comparisons between the MMF and CYC subsets

^b^These *p*-values are for comparisons between the RTX and no RTX subsets

^c^Asian race includes East Asian and South Asian

^d^Other constitutes either heterogeneous race (≥ 2 races were reported), or race was not reported

^e^Mixed class denotes class V concomitant with either class III or IV

Hematuria and pyuria were found in 84% and 45% of all patients, respectively. There was a significant difference in the presence of hematuria between class IV and pure class V (97% vs. 56%, *p*_adj_ = 0.004), but no differences were found for the presence of pyuria (Table 2).

**Table 2:** Laboratory test data, presence of any stage of acute kidney injury (AKI), and presence of hypertension (HTN) at the time of pediatric lupus nephritis (pLN) diagnosis for the composite 108-patient cohort with stratification by biopsy class. For categorical values, number of affected patients out of the total number of patients for which data were available and the corresponding percentages are provided. For numerical data, median values, their corresponding lower quartiles (Q1) and upper quartiles (Q3), and number of patients for which data were available are provided. Please see the footnotes for the descriptions of groupings leading to statistical significance

Lab value	Normal range	All patients	Class III	Class IV	Mixed class^a^	Pure class V	*p*-value
Hematuria present^b^		75/89 (84%)	16/19 (84%)	31/32 (97%)	18/20 (90%)	10/18 (56%)	0.002*^f^
Pyuria present^c^		40/89 (45%)	7/19 (37%)	17/32 (53%)	12/20 (60%)	4/18 (22%)	0.074
UPCR (mg/mg)	< 0.2						0.009*^g^
UPCR > 0.2– ≤ 2		34/81 (42%)	13/17 (76%)	7/28 (25%)	6/16 (38%)	8/20 (40%)	
UPCR > 2		47/81 (58%)	4/17 (24%)	21/28 (75%)	10/16 (63%)	12/20 (60%)	
Albumin (g/dL)	3.4–5.2	2.8 (2.3–3.2) *n* = 106	3.2 (2.6–3.6) *n* = 18	2.7 (2.3–3.2) *n* = 37	2.4 (2.0–3.1) *n* = 27	3.0 (2.3–3.3) *n* = 24	0.01*^h^
C3 (mg/dL)	90–154	53 (36–83) *n* = 92	52 (38–81) *n* = 19	44 (33–71) *n* = 33	43 (34–73) *n* = 19	78 (53–110) *n* = 21	0.057
eGFR (mL/min/1.73 m^2^)	≥ 90	90 (61–113) *n* = 106	93 (81–114) *n* = 19	62 (44–82) *n* = 37	90 (64–111) *n* = 27	111 (94–130) *n* = 23	< 0.001*^i^
AKI present^d^		47/106 (44%)	5/19 (26%)	27/37 (73%)	11/27 (41%)	4/23 (17%)	< 0.001*^j^
HTN present^e^		48/104 (46%)	5/19 (26%)	25/37 (68%)	13/25 (52%)	5/23 (22%)	0.001*^k^

Abbreviations: *AKI, *acute kidney injury; *eGFR, *estimated glomerular function rate; *HTN, *hypertension; *UPCR, *urine protein-to-creatinine ratio

^a^Mixed class denotes class V concomitant with either class III or IV

^b^Hematuria was defined as urine RBC ≥ 3/high powered field

^c^Pyuria was defined as urine WBC ≥ 5/high powered field

^d^AKI staging definitions used are from the KDIGO 2012 AKI guidelines [11]

^e^HTN was defined as either systolic blood pressure (SBP) or diastolic blood pressure (DBP) ≥ 95th

percentile for patients aged < 13 years and SBP ≥ 130 or DBP ≥ 80 for patients aged ≥ 13 years [17]

^f^Class IV vs. pure class V (*p*_adj_ = 0.004)

^g^Class III vs. class IV (*p*_adj_ = 0.01)

^h^Class III vs. class IV (*p*_adj_ = 0.047), class III vs. mixed class (*p*_adj_ = 0.012)

^i^Class IV vs. class III (*p*_adj_ = 0.01); class IV vs. pure class V (*p*_adj_ < 0.001)

^j^Class IV vs. class III (*p*_adj_ = 0.035); class IV vs. pure class V (*p*_adj_ < 0.001)

^k^Class IV vs. class III (*p*_adj_ = 0.024); class IV vs. pure class V (*p*_adj_ = 0.007)

Regarding proteinuria, 42% had sub-nephrotic range proteinuria (highest incidence in class III at 76%) and 58% had nephrotic-range proteinuria (highest incidence in class IV at 75%). Class IV had significantly higher degrees of proteinuria than Class III (*p*_adj_ = 0.0095). The median serum albumin level for all patients was 2.8 g/dL, with the lowest level in mixed class (2.4 g/dL). The median serum albumin level in class III (3.2 g/dL) was significantly higher compared to class IV (2.7 g/dL, *p*_adj_ = 0.047) and mixed class (2.4 g/dL, *p*_adj_ = 0.012) (Table 2).

The median eGFR for all patients was 90 mL/min/1.73 m^2^, with the lowest eGFR in class IV (62 mL/min/1.73 m^2^). The eGFR for class IV was significantly lower compared to class III (93 mL/min/1.73 m^2^, *p*_adj_ = 0.009) and pure class V (111 mL/min/1.73 m^2^, *p*_adj_ < 0.001). AKI was seen in 44% of patients, with the highest incidence in class IV (73%). The AKI incidence rate for class IV was significantly higher compared to class III (26%, *p*_adj_ = 0.035) and pure class V (17%, *p*_adj_ < 0.001) (Table 2).

Stage 3 AKI was present for six patients with class IV and two patients with mixed class. Three of these eight patients, all with class IV, required acute kidney support therapy (KST), and two patients remained dialysis-dependent. A third patient with class IV required acute KST after a pLN relapse and had resultant advanced CKD after KST liberation. These three patients received kidney transplants at 60, 30, and 54 months, respectively.

HTN was present in 46% of all patients, with the highest incidence in class IV (68%). The HTN incidence rate for class IV (68%) was significantly higher than class III (26%, *p*_adj_ = 0.024) and pure class V (22%, *p*_adj_ = 0.0071) (Table 2).

**Fig. 2:**
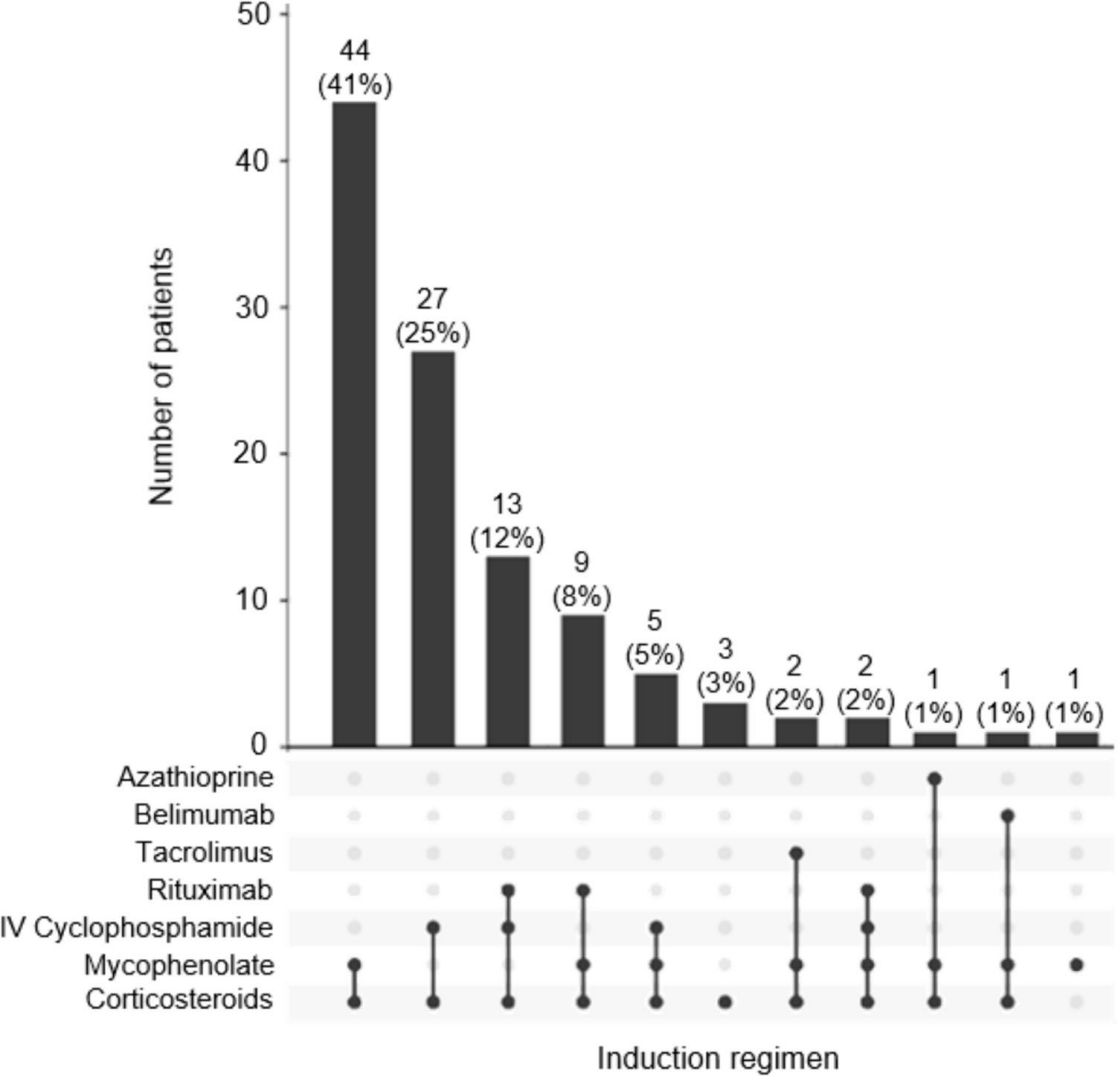
Initial therapy regimens received in the composite 108-patient cohort. Initial therapy is defined as medications received ≤ 3 months from the time of initial pediatric lupus nephritis biopsy. Regimens that consist of multiple medications are illustrated using an UpSet plot. The number of patients that received each treatment regimen and the corresponding percentage out of the total 108 patients is provided. Corticosteroids include both IV and oral formulations. IV cyclophosphamide encompasses both EuroLupus and the National Institutes of Health dosing protocols. Mycophenolate includes both mycophenolate mofetil and mycophenolic acid

The two most common initial therapy regimens were CS with MMF (41%) and CS with CYC (25%). Less common regimens were CS monotherapy (3%) and MMF monotherapy (1%). RTX was used as adjunctive therapy in 22% of patients (Fig. 2).

### MMF (without RTX) vs. CYC (without RTX) subsets (51 patients)

A total of 51 patients received CS and either MMF (33 patients) or CYC (18 patients) without RTX for initial therapy. CYC was used more often in class IV than MMF (67% vs. 24%, *p*_adj_ = 0.0076), whereas no patients with pure class V received CYC (0% vs. 36%, *p*_adj_ = 0.0068) (Table 1).

Compared to MMF, patients in the CYC subset had significantly higher rates of AKI (61% vs. 18%, *p* = 0.0042) at time of biopsy as well as a trend towards lower eGFR (76 vs. 107 mL/min/1.73 m^2^, *p* = 0.057). Otherwise, there were no significant differences between the MMF and CYC subsets regarding the presence of hematuria or pyuria, degree of proteinuria, serum albumin level, eGFR, or presence of advanced CKD or HTN at any timepoint through 24-month follow-up (Table 3).

**Table 3:** Laboratory test data, presence of any stage of acute kidney injury (AKI) at time of diagnosis, presence of chronic kidney disease (CKD) at ≥ 6 months of follow-up, and presence of hypertension (HTN) at time of diagnosis and at ≥ 6 months of follow-up for patients who received: corticosteroids (CS) and mycophenolate (MMF) (labeled MMF); CS and IV cyclophosphamide (CYC) (labeled CYC); CS and either MMF or CYC without rituximab (RTX) (labeled No RTX); and CS and either MMF or CYC with RTX (labeled RTX). For categorical values, number of affected patients out of the total number of patients for which data were available and the corresponding percentages are provided. For numerical data, median values, their corresponding lower quartiles (Q1) and upper quartiles (Q3), and number of patients for which data were available are provided

	MMF (*n* = 33)	CYC (*n* = 18)	*p*-value^a^	No RTX (*n* = 51)	RTX (*n* = 20)	*p*-value^b^
**Time of diagnosis**						
Hematuria present^c^	23/28 (82%)	17/17 (100%)	0.14	40/45 (89%)	18/20 (90%)	1.00
Pyuria present^d^	7/28 (25%)	8/17 (47%)	0.19	15/45 (33%)	14/20 (70%)	0.008*
UPCR (mg/mg)			0.77			1.00
UPCR > 0.2– ≤ 2	15/33 (45%)	7/18 (39%)		22 (43%)	9/20 (45%)	
UPCR > 2	18/33 (55%)	11/18 (61%)		29 (57%)	11/20 (55%)	
Albumin (g/dL)	3.1 (2.5–3.5) *n* = 32	2.9 (2.2–3.2) *n* = 18	0.088	3.0 (2.4–3.3) *n* = 50	2.5 (2.1–2.9) *n* = 20	0.051
C3 (mg/dL)	81 (42–109) *n* = 33	42 (32–55) *n* = 16	< 0.001*	55 (39–86) *n* = 49	52 (31–66) *n* = 20	0.10
eGFR (mL/min/1.73 m^2^)	107 (91–123) *n* = 33	76 (62–99) *n* = 18	0.057	96 (74–121) *n* = 51	65 (54–97) *n* = 20	0.068
AKI present^e^	6/33 (18%)	11/18 (61%)	0.004*	16/51 (31%)	13/20 (65%)	0.015*
HTN present^f^	14/19 (42%)	12/17 (71%)	0.078	26/50 (52%)	11/20 (55%)	1.00
**6-month follow-up**						
Hematuria present^c^	11/21 (52%)	10/15 (67%)	0.50	21/36 (58%)	11/18 (61%)	1.00
Pyuria present^d^	2/21 (10%)	1/15 (7%)	1.00	3/36 (8%)	2/18 (11%)	1.00
UPCR (mg/mg)			0.30			0.40
UPCR ≤ 0.2	12/23 (52%)	4/15 (27%)		17/39 (44%)	6/18 (33%)	
UPCR > 0.2– ≤ 2	10/23 (44%)	10/15 (66%)		20/39 (51%)	9/18 (50%)	
UPCR > 2	1/23 (4%)	1/15 (7%)		2/39 (5%)	3/18 (17%)	
Albumin (g/dL)	3.9 (3.8–4.4) *n* = 31	4.0 (3.8–4.2) *n* = 16	0.82	4.0 (3.8–4.4) *n* = 47	4.2 (3.9–4.4) *n* = 20	0.17
C3 (mg/dL)	102 (88–132) *n* = 29	118 (81–128) *n* = 16	0.60	103 (86–131) *n* = 45	118 (89–132) *n* = 19	0.77
eGFR (mL/min/1.73 m^2^)	107 (93–119) *n* = 30	100 (87–106) *n* = 16	0.076	104 (92–117) *n* = 46	102 (82–116) *n* = 20	0.80
CKD present^g^			0.70			0.069
Stage 1	24/29 (83%)	12/16 (75%)		36/45 (80%)	11/20 (55%)	
Stages 2–5	5/29 (17%)	4/16 (25%)		9/45 (20%)	9/20 (45%)	
HTN present^f^	8/31 (26%)	4/16 (25%)	1.00	12/47 (26%)	4/20 (20%)	0.76
**12-month follow-up**						
Hematuria present^c^	6/21 (29%)	7/14 (50%)	0.29	13/35 (37%)	11/20 (55%)	0.26
Pyuria present^d^	2/21 (10%)	2/14 (14%)	1.00	4/35 (11%)	2/20 (10%)	1.00
UPCR (mg/mg)			0.79			0.10
UPCR ≤ 0.2	13/22 (59%)	10/14 (72%)		23/36 (64%)	9/17 (53%)	
UPCR > 0.2– ≤ 2	6/22 (27%)	2/14 (14%)		8/36 (22%)	8/17 (47%)	
UPCR > 2	3/22 (14%)	2/14 (14%)		5/36 (14%)	0/17 (0%)	
Albumin (g/dL)	4.1 (4.0–4.6) *n* = 23	4.4 (4.1–4.4) *n* = 16	0.85	4.3 (4.0–4.5) *n* = 39	4.2 (4.0–4.6) *n* = 19	0.86
C3 (mg/dL)	105 (86–133) *n* = 25	108 (85–126) *n* = 15	0.48	106 (85–128) *n* = 40	117 (102–134) *n* = 19	0.10
eGFR (mL/min/1.73 m^2^)	104 (96–126) *n* = 27	102 (92–121) *n* = 17	0.27	103 (93–123) *n* = 44	92 (75–115) *n* = 19	0.067
CKD present^g^			0.40			0.17
Stage 1	24/27 (89%)	13/17 (76%)		37/44 (84%)	12/18 (67%)	
Stages 2–5	3/27 (11%)	4/17 (24%)		7/44 (16%)	6/18 (33%)	
HTN present^f^	3/26 (12%)	2/17 (12%)	1.00	5/43 (12%)	6/20 (30%)	0.088
**24-month follow-up**						
Hematuria present^c^	5/18 (28%)	3/8 (38%)	0.67	8/26 (31%)	8/15 (53%)	0.19
Pyuria present^d^	3/18 (17%)	1/8 (13%)	1.00	4/26 (15%)	3/15 (20%)	0.69
UPCR (mg/mg)			0.17			0.054
UPCR ≤ 0.2	8/14 (57%)	4/8 (50%)		12/22 (55%)	11/17 (65%)	
UPCR > 0.2– ≤ 2	1/14 (7%)	3/8 (38%)		4/22 (18%)	6/17 (35%)	
UPCR > 2	5/14 (36%)	1/8 (12%)		6/22 (27%)	0/17 (0%)	
Albumin (g/dL)	4.1 (4.0–4.4) *n* = 17	4.5 (4.3–4.8) *n* = 9	0.099	4.3 (4.0–4.7) *n* = 26	4.4 (4.1–4.5) *n* = 16	0.91
C3 (mg/dL)	105 (91–128) *n* = 21	110 (80–124) *n* = 9	0.73	106 (87–127) *n* = 30	118 (102–126) *n* = 16	0.59
eGFR (mL/min/1.73 m^2^)	103 (94–127) *n* = 21	105 (98–121) *n* = 9	0.87	104 (95–125) *n* = 30	108 (97–134) *n* = 16	0.40
CKD present^g^			1.00			1.00
Stage 1	17/21 (81%)	8/9 (89%)		25/30 (83%)	14/16 (88%)	
Stages 2–5	4/21 (19%)	1/9 (11%)		5/30 (17%)	2/16 (12%)	
HTN present^f^	6/21 (29%)	0/7 (100%)	0.29	6/28 (21%)	1/16 (6%)	0.39

Abbreviations: *AKI, *acute kidney injury; *CKD, *chronic kidney disease; *eGFR, *estimated glomerular function rate; *HTN, *hypertension; *RTX, *rituximab; *UPCR, *urine protein-to-creatinine ratio

^a^These *p*-values are for comparisons between the MMF and CYC subset

^b^These *p*-values are for comparisons between the RTX and no RTX subset

^c^Hematuria was defined as urine RBC ≥ 3/high powered field

^d^Pyuria was defined as urine WBC ≥ 5/high powered field

^e^AKI staging definitions used are from the KDIGO 2012 AKI guidelines [11]

^f^HTN was defined as either systolic blood pressure (SBP) or diastolic blood pressure (DBP) ≥ 95th percentile for patients aged < 13 years and SBP ≥ 130 or DBP ≥ 80 for patients aged ≥ 13 years [17]

^g^CKD staging definitions used are from the KDIGO 2024 CKD guidelines [14]

There were no significant differences in CR or CR + PR at any timepoint between patients who received MMF and CYC when biopsy classes were included together or stratified separately. No comparisons could be made for class III at 24 months since no data were available for those who received CYC, or for pure class V at any timepoint since those patients only received MMF (Table 4).

**Table 4:** Percentage of patients who achieved complete response (CR) and any response (CR or partial response (PR)) at 6-, 12-, and 24-month follow-ups after receiving initial therapy with corticosteroids (CS) and either mycophenolate (MMF) or IV cyclophosphamide (CYC) regardless of pediatric lupus nephritis (pLN) biopsy class (labeled as “all”) and stratified by pLN biopsy class. Number of patients who achieved the specified response out of the total number of patients for which data were available and the corresponding percentages are provided. Dashed lines denote that there were no available data for that time point. *P-values* are listed as not applicable (N/A) when data were not available for comparison or the comparison was between data with identical values

		Complete response			Any response (CR or PR)		
**pLN class**	**Initial therapy**	6 months	12 months	24 months	6 months	12 months	24 months
**All**	MMF	10/23 (43%)	13/22 (59%)	7/14 (50%)	20/23 (87%)	18/22 (82%)	7/14 (50%)
	CYC	4/15 (27%)	10/14 (71%)	4/8 (50%)	13/15 (87%)	12/14 (86%)	7/8 (88%)
	*p*-value	0.33	0.50	1.00	1.00	1.00	0.17
**III**	MMF	2/5 (40%)	5/5 (100%)	3/4 (75%)	3/5 (60%)	5/5 (100%)	3/4 (75%)
	CYC	1/1 (100%)	2/2 (100%)	-	1/1 (100%)	2/2 (100%)	-
	*p*-value	1.00	N/A	-	1.00	N/A	-
**IV**	MMF	2/5 (40%)	2/4 (50%)	1/3 (33%)	5/5 (100%)	3/4 (75%)	1/3 (33%)
	CYC	3/11 (27%)	7/10 (70%)	4/6 (67%)	10/11 (91%)	8/10 (80%)	6/6 (100%)
	*p*-value	1.00	0.58	0.52	1.00	1.00	0.083
**Mixed class**^**a**^	MMF	3/4 (75%)	3/5 (60%)	1/2 (50%)	4/4 (100%)	4/5 (80%)	1/2 (50%)
	CYC	0/3 (0%)	1/2 (50%)	0/2 (0%)	2/3 (67%)	2/2 (100%)	1/2 (50%)
	*p*-value	0.14	1.00	1.00	0.43	1.00	1.00
**Pure class V**	MMF	3/9 (33%)	3/8 (38%)	2/5 (40%)	8/9 (89%)	6/8 (75%)	2/5 (40%)
	CYC^b^	-	-	-	-	-	-
	*p*-value	N/A	N/A	N/A	N/A	N/A	N/A

Abbreviations: *CYC, *IV cyclophosphamide; *CR, *complete response; *MMF, *mycophenolate; *N/A, *not applicable; *PR, *partial response; *pLN, *pediatric lupus nephritis

^a^Mixed class denotes class V concomitant with either class III or IV

^b^No patients with pure class V pLN received CYC

### RTX vs. no RTX subsets (71 patients)

A total of 71 patients received CS and either MMF or CYC with RTX (*n* = 20) or without RTX (*n* = 51) for initial therapy. There were no significant differences in demographics or characteristics between these patient subsets (Table 1).

Patients who received RTX as part of their initial therapy had significantly higher rates of pyuria (70% vs. 33%, *p* = 0.0078) and AKI (65% vs. 31%, *p* = 0.015) at biopsy; they also had trends towards lower serum albumin level (2.5 vs. 3.0 g/dL, *p* = 0.051) and lower eGFR (65 vs. 96 mL/min/1.73 m^2^, *p* = 0.068) at biopsy. At 24 months, the RTX subset had less proteinuria and no nephrotic-range proteinuria (0% vs. 14%, *p* = 0.054). There were no significant differences in presence of hematuria, advanced CKD, or HTN at any timepoint (Table 3).

When evaluating all pLN classes together, the RTX subset had significantly higher CR + PR at 24 months compared to non-RTX (100% vs. 64%, *p* = 0.012), but no significant differences existed for CR + PR at 6 or 12 months or in CR at any timepoint. When stratifying by biopsy class, the only significant difference seen was class IV treated without RTX had increased CR at 12 months compared to RTX treatment (64% vs. 0%, *p* = 0.033) (Table 5).

**Table 5:** Percentage of patients who achieved complete response (CR) and any response (CR or partial response [PR]) at 6-, 12-, and 24-month follow-ups after receiving rituximab (RTX) or no RTX as part of their initial therapy along with corticosteroids and either mycophenolate or IV cyclophosphamide regardless of pediatric lupus nephritis (pLN) biopsy class (labeled as “all”) and stratified by pLN biopsy class. Number of patients who achieved the specified response out of the total number of patients for which data were available and the corresponding percentages are provided

		Complete response			Any response (CR or PR)		
**pLN class**	**Initial therapy**	6 months	12 months	24 months	6 months	12 months	24 months
**All**	No RTX	15/39 (38%)	23/36 (64%)	11/22 (50%)	34/39 (87%)	30/36 (83%)	14/22 (64%)
	RTX	5/18 (28%)	6/16 (38%)	11/16 (69%)	13/18 (72%)	11/16 (69%)	16/16 (100%)
	*p*-value	0.56	0.077	0.52	0.26	0.28	0.012*
**III**	No RTX	3/6 (50%)	7/7 (100%)	3/4 (75%)	4/6 (67%)	7/7 (100%)	3/4 (75%)
	RTX	4/7 (57%)	3/6 (50%)	4/6 (67%)	6/7 (86%)	5/6 (83%)	6/6 (100%)
	*p*-value	1.00	0.070	1.00	0.56	0.46	0.40
**IV**	No RTX	5/16 (31%)	9/14 (64%)	5/9 (56%)	15/16 (94%)	11/14 (79%)	7/9 (78%)
	RTX	1/5 (20%)	0/4 (0%)	3/4 (75%)	3/5 (60%)	3/4 (75%)	4/4 (100%)
	*p*-value	1.00	0.033*	1.00	0.13	1.00	1.00
**Mixed class**^a^	No RTX	3/7 (43%)	4/7 (57%)	1/4 (25%)	6/7 (86%)	6/7 (86%)	2/4 (50%)
	RTX	0/3 (0%)	2/2 (100%)	1/3 (33%)	2/3 (67%)	2/2 (100%)	3/3 (100%)
	*p*-value	0.48	0.50	1.00	1.00	1.00	0.43
**Pure class V**	No RTX	4/10 (40%)	3/8 (38%)	2/5 (40%)	9/10 (90%)	6/8 (75%)	2/5 (40%)
	RTX	0/3 (0%)	1/4 (25%)	3/3 (100%)	2/3 (67%)	1/4 (25%)	3/3 (100%)
	*p*-value	0.50	1.00	0.20	0.42	0.22	0.20

Abbreviations: *CR*, complete response; *PR*, partial response; *pLN*, pediatric lupus nephritis; *RTX*, rituximab

^a^Mixed class denotes class V concomitant with either class III or IV

These data were also analyzed after stratifying by initial therapy received with either MMF (MMF vs. MMF and RTX) or CYC (CYC vs. CYC and RTX), and these results can be found in Supplemental Tables 1–4.

### Overall response rates

**Fig. 3:**
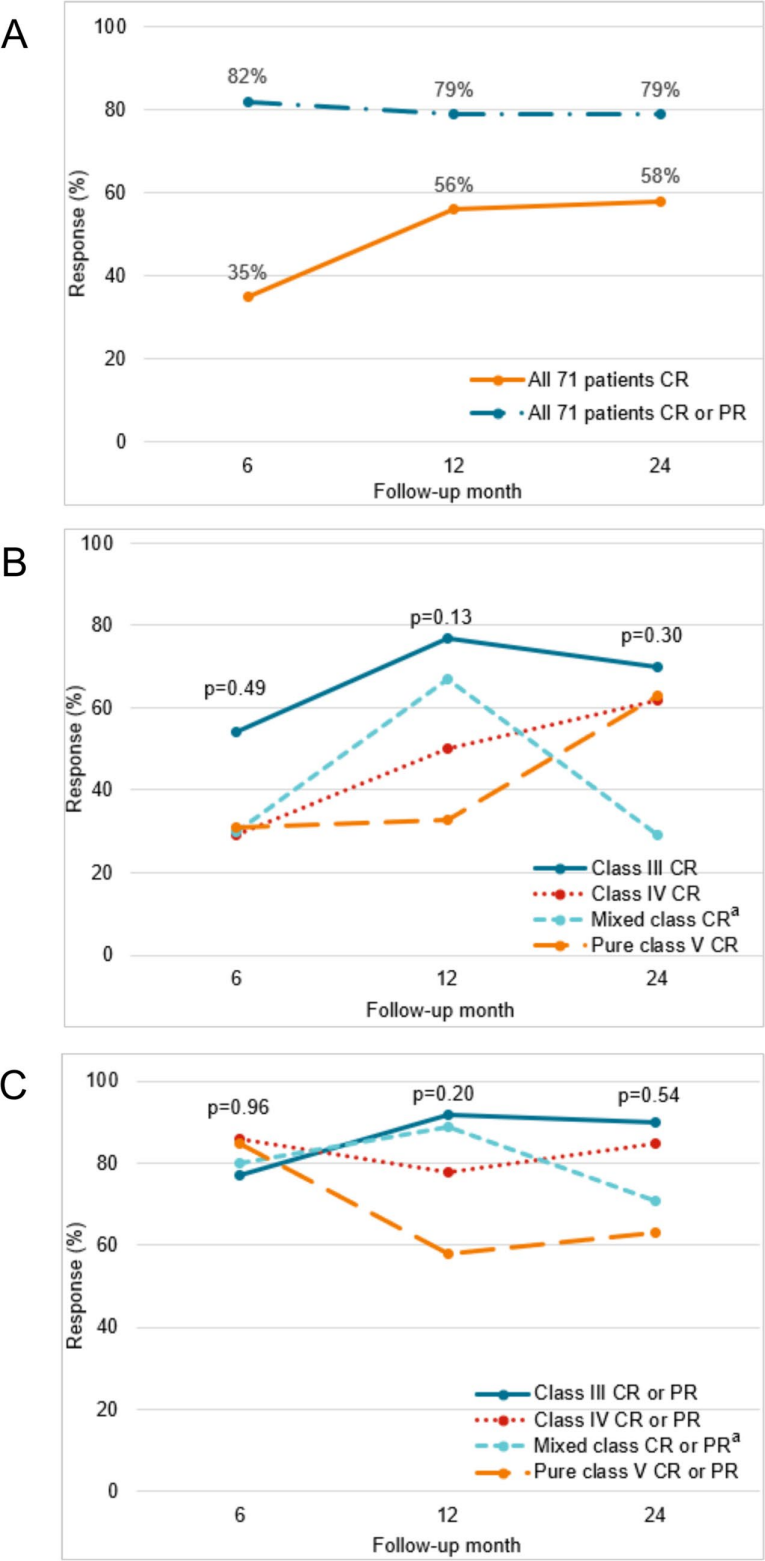
Percentage of patients in the 71-patient subset at 6-, 12-, and 24-month follow-ups regardless of initial therapy received who achieved **A** complete response (CR) and any response (CR or partial response [PR]) regardless of pediatric lupus nephritis (pLN) biopsy class; **B** CR stratified by pLN biopsy class; and **C** CR or PR stratified by pLN biopsy class. *P*-values are provided for each time point within the graphs for **B** and **C**. Total number of patients for which data was available at each timepoint: 57 at 6 months, 52 at 12 months, and 38 at 24 months. Abbreviations: CR, complete response; PR, partial response. ^a^Mixed class denotes class V concomitant with either class III or IV

For all 71 patients, regardless of biopsy class or initial therapy, overall CR rates at 6 and 24 months were 35% and 58%, respectively, and CR + PR rates at 6 and 24 months were 82% and 79%, respectively (Fig. 3A). There were no significant differences in CR (Fig. 3B) or CR + PR (Fig. 3C) at any timepoint when stratified by biopsy class regardless of initial therapy. Class III had the highest CR and CR + PR at 24 months at 70% and 90%, respectively. The lowest CR and CR + PR at 24 months were 29% (mixed class) and 63% (pure class V), respectively (Fig. 3B and C).

### Secondary outcomes

The percentages of patients still receiving CS were 93% (at 6 months), 80% (at 12 months), and 51% (at 24 months) of all 71 patients. There were no significant differences in CS usage at any timepoint between MMF vs. CYC and RTX vs. non-RTX subsets (Supplemental Table 5).

At least one CS side effect was reported within 24 months by 40 of the 71 patients (56%), and seven of the 71 patients (10%) reported experiencing ≥ 2 side effects. The most common CS side effect was Cushingoid features (36 patients, 51%). Uncommon, but severe, side effects included insulin-dependent diabetes mellitus (2 patients, 3%), osteopenia with fractures (2 patients, 3%), cataracts (1 patient, 1%), and avascular necrosis (1 patient, 1%).

Infections were experienced by 13% (at 6 months), 13% (at 12 months), and 21% (at 24 months) of all 71 patients with no significant differences in infection rates at any timepoint between MMF vs. CYC and RTX vs. non-RTX subsets (Supplemental Table 6). The most common infections were viral upper respiratory tract infections (URIs) and viral gastroenteritis. Uncommon, but severe, infections included bacterial endocarditis (1 patient, CYC and RTX); varicella zoster (2 patients, CYC and RTX); septic arthritis (1 patient, MMF and RTX); and bacterial sepsis and *Clostridium difficile* enterocolitis ultimately leading to death (1 patient, MMF).

One patient receiving MMF became pregnant with an obstetric course complicated by pre-eclampsia.

pLN relapse affected 27% (at 6 months), 17% (at 12 months), and 13% (at 24 months) of all 71 patients. There was a significant increase in 6-month pLN relapse rate in the RTX cohort compared to non-RTX (50% vs. 17%, *p* = 0.014), but no significant differences were found at 12 or 24 months or at any timepoint between MMF and CYC (Supplemental Table 7).

## Discussion

This multicenter, prospective study provides much needed evidence in US pLN patients for efficacy and safety data of MMF versus CYC, with or without RTX, as an adjunctive agent for initial therapy. As evidenced by the variability of initial regimens used (Fig. 2), pediatric patients have not been receiving standardized care, and even children receiving typical regimens with MMF or CYC still have sub-optimal response rates. Additionally, CS use and side effect burden were high.

In our study, MMF demonstrated similar efficacy to CYC for pLN initial therapy, with comparable kidney outcomes and response rates at 6-, 12-, and 24-month follow-ups. One caveat to consider is that the CYC subset presented with lower eGFR, indicating more significant disease. We did evaluate response rates in each biopsy class to gain further insight into MMF’s efficacy in more severe disease. Although patient numbers were small, there were no significant differences in response rates at any timepoint of follow-up between MMF and CYC for each biopsy class. Moreover, rates of pLN relapse were not significantly different at any timepoint between MMF and CYC.

Our results are consistent with other pLN studies regarding outcomes when MMF is used for initial therapy. One retrospective study evaluated 6-, 12-, and 24-month kidney outcomes in 31 Taiwanese patients and found similar response rates between MMF and CYC but improved eGFR with MMF [21]. A combined prospective and retrospective study assessed 12-month kidney outcomes in 79 patients from German and Austrian centers and found no differences in eGFR or response rates between MMF and CYC [6]. Another prospective study evaluated kidney outcomes in 51 patients in the United Kingdom and found no significant differences between MMF and CYC at 4–8 and 10–14 months [7].

When RTX was given as part of initial therapy, CR + PR and the incidence of nephrotic-range proteinuria were improved at 24 months, but not at 6 or 12 months, suggesting RTX may be more beneficial in helping to achieve long-term response rather than in inducing an earlier response. Interestingly, pLN relapses were significantly increased in the RTX patient subset at 6 months but not at 12 or 24 months. The RTX cohort presented with increased rates of AKI and lower serum albumin levels, indicating more severe disease that may inherently be more refractory to treatment. Additionally, different polymorphisms lead to variations in the phenotypic expression of Fcγ receptors on immune cells, of which a part of RTX crosslinks to, so these differences in receptors affect responsiveness to RTX [22–24].

There are few studies evaluating the efficacy of RTX for pLN initial therapy, and all are retrospective. In a 44-patient cohort in India, 36-month kidney outcomes were improved when RTX was included with CS and either MMF or CYC for initial therapy [25]. In a 12-patient cohort in France, 6- and 12-month CR + PR was achieved for all patients after receiving CS, MMF, and RTX for initial therapy [26].

Response rates in our study are similar to those previously described. In two international, retrospective studies, 53% of a 382-patient cohort and 48–79% of a 248-patient cohort had achieved CR at 24-month follow-up [3, 27]. A retrospective study from the US with 176 patients found 40–60% remained in CR at 24 months [28].

No particular initial therapy regimen was associated with earlier CS weaning. CS dosing and treatment length were variable across all subsets, suggesting that individual provider practice influences CS dosing more than disease characteristics [29]. A large majority of patients experienced at least one CS side effect, most commonly Cushingoid features. These physical changes compound the distressing psychosocial effects from chronic disease, especially during adolescence [30, 31]. CS can also affect growth potential [32], additionally contributing to poor body image. Although rare, patients experienced cataracts, avascular necrosis, and osteopenia with fractures, highlighting the need for judicious CS use.

The addition of RTX to initial therapy was not associated with increased infection rates. Infections, including influenza and chlamydia, underscore the importance of risk-mitigation counseling regarding vaccination and safe-sex practices. The importance of safe-sex counseling is additionally highlighted by one patient receiving MMF becoming pregnant. SLE confers increased morbidity for both the fetus and pregnant patient [33], and teratogenic medications add additional risk to the fetus. Therefore, assessing patients’ current and future reproductive goals is imperative.

The majority of patients were assigned female sex at birth, African American, and aged 14–17 years, mirroring the known trends in SLE [34]. Class IV and V mixed presented with more severe disease, and mixed class had the lowest CR (29%) and the second-lowest CR + PR (71%) at 24 months. These data align with the more severe presentations and courses of class IV and V mixed [2, 3]. Pure class V presented with the highest eGFR but increased rates of nephrotic-range proteinuria. This proteinuria appears to be more refractory to treatment and likely contributed to pure class V having the lowest 24-month CR (63%), similar to another study that found a 24-month CR of 74% in 56 patients with pure class V [35].

The timeframe of our study was such that agents, like belimumab and calcineurin inhibitors, were not widely used in our patients. Belimumab has been shown to decrease proteinuria and rate of LN relapses when used in conjunction with MMF or CYC [36]. Additionally, the addition of voclosporin to MMF and low-dose CS therapy has demonstrated improved CR at 6- and 12-months [37] and continued safety and efficacy up to three years of treatment [38]. An emerging therapy is second-generation anti-CD20 monoclonal antibodies, and for adult patients with LN, addition of obinutuzumab to standard therapy has demonstrated improved rates of complete remission [39]. These new treatment options offer promise in the treatment of pLN and are included in the ACR 2024 LN guidelines. The recommendation is to initiate a triple therapy immunosuppression regimen at the time of diagnosis for patients with class III, class IV, mixed class, and pure class V associated with a UPCR > 1 mg/mg [5]. As pediatric rheumatologists and nephrologists begin to use these agents, we will benefit from collecting prospective, real-world data in order to determine optimal management strategies for our patients.

The largest limitation of our study is inconsistent data collection due to variable follow-up given its prospective nature. Because of the limited sample size and sparse outcome counts, treatment effect estimates are imprecise and potentially unstable. The study is therefore likely underpowered, and further studies are needed to establish clinically meaningful differences in outcomes. Although cohorts had closely matched demographics and investigation results at presentation, non-statistically significant, but potentially clinically relevant, differences in eGFR exist in the MMF vs. CYC and RTX vs. non-RTX subsets, prior to respective treatments. CYC and RTX dosing regimens were based on a center’s practice and not standardized, and data for patients who received EuroLupus and NIH dosing regimens of CYC were aggregated as patient numbers were too small for separate analyses. Baseline or post-RTX CD19/CD20 counts were not captured, so data regarding B-cell depletion and reconstitution are unknown. We acknowledge that 12- and 24-month outcomes are related to initial and maintenance therapies, but given the complexity of including maintenance therapy into outcomes, this was not performed.

Our study has several strengths. As previously mentioned, this study is one of the few prospective pLN studies comparing MMF to CYC and the first to evaluate RTX as an adjunct to initial therapy. There was geographical diversity between the 8 pediatric centers that participated, and patients were a representative sample of the pLN population cared for in the US.

In conclusion, our study, one of the first prospective examinations of pLN, supports the use of MMF as an initial agent in LN. Additionally, RTX use during initial therapy may lead to improved long-term outcomes, without increasing infection rates. However, response rates were not optimal, and large variability in initial therapy and CS regimens were found. pLN and its potential sequelae, including advanced CKD with potential need for dialysis and kidney transplantation, are too high stakes to continue on without high-level evidence. This study highlights the need for development of evidence-based, standardized treatment regimens for pLN, rather than those solely based on adult-centered clinical trials.

## Supplementary Information

Below is the link to the electronic supplementary material.

ESM 1 Graphical abstract (PPTX 107 KB)

ESM 2 (DOCX 55.9 KB)

## Data Availability

The datasets generated and/or analyzed during the current study are available from the corresponding author on reasonable request.

## References

[1] Anders HJ, Saxena R, Zhao MH, Parodis I, Salmon JE, Mohan C (2020) Lupus nephritis. Nat Rev Dis Primers 6:7. 10.1038/s41572-019-0141-9 31974366 10.1038/s41572-019-0141-9

[2] Oni L, Wright RD, Marks S, Beresford MW, Tullus K (2021) Kidney outcomes for children with lupus nephritis. Pediatr Nephrol 36:1377–1385. 10.1007/s00467-020-04686-1 32725543 10.1007/s00467-020-04686-1 PMC8084759

[3] De Mutiis C, Wenderfer SE, Basu B et al (2023) International cohort of 382 children with lupus nephritis - presentation, treatment and outcome at 24 months. Pediatr Nephrol 38:3699–3709. 10.1007/s00467-023-06018-5 37221349 10.1007/s00467-023-06018-5

[4] Kidney Disease: Improving Global Outcomes Lupus Nephritis Work Group (2024) KDIGO 2024 clinical practice guideline for the management of lupus nephritis. Kidney Int 105:S1–S69. 10.1016/j.kint.2023.09.002 38182286 10.1016/j.kint.2023.09.002

[5] Sammaritano LR, Askanase A, Bermas BL et al (2025) American College of Rheumatology (ACR) guideline for the screening, treatment, and management of lupus nephritis. Arthritis Care Res 77:1045–1065. 10.1002/acr.25528 10.1002/acr.25528 40127995

[6] Suhlrie A, Hennies I, Gellermann J et al (2020) Twelve-month outcome in juvenile proliferative lupus nephritis: results of the German registry study. Pediatr Nephrol 35:1235–1246. 10.1007/s00467-020-04501-x 32193650 10.1007/s00467-020-04501-x

[7] Smith E, Al-Abadi E, Armon K et al (2019) Outcomes following mycophenolate mofetil versus cyclophosphamide induction treatment for proliferative juvenile-onset lupus nephritis. Lupus 28:613–620. 10.1177/0961203319836712 30871425 10.1177/0961203319836712

[8] Vazzana KM, Daga A, Goilav B et al (2021) Principles of pediatric lupus nephritis in a prospective contemporary multi-center cohort. Lupus 30:1660–1670. 10.1177/09612033211028658 34219529 10.1177/09612033211028658 PMC10461610

[9] Bajema IM, Wilhelmus S, Alpers CE et al (2018) Revision of the International Society of Nephrology/Renal Pathology Society classification for lupus nephritis: clarification of definitions, and modified National Institutes of Health activity and chronicity indices. Kidney Int 93:789–796. 10.1016/j.kint.2017.11.023 29459092 10.1016/j.kint.2017.11.023

[10] Hessey E, Ali R, Dorais M et al (2017) Evaluation of height-dependent and height-independent methods of estimating baseline serum creatinine in critically ill children. Pediatr Nephrol 32:1953–1962. 10.1007/s00467-017-3670-z 28523356 10.1007/s00467-017-3670-z

[11] Kidney Disease: Improving Global Outcomes (KDIGO) Acute Kidney Injury Work Group (2012) KDIGO clinical practice guidelines for acute kidney injury. Kidney Int Suppl 2:1–138. 10.1159/000339789

[12] Ng DK, Pierce CB (2021) Kidney disease progression in children and young adults with pediatric CKD: epidemiologic perspectives and clinical applications. Semin Nephrol 41:405–415. 10.1016/j.semnephrol.2021.09.002 34916001 10.1016/j.semnephrol.2021.09.002 PMC8694646

[13] Pierce CB, Munoz A, Ng DK, Warady BA, Furth SL, Schwartz GJ (2021) Age- and sex-dependent clinical equations to estimate glomerular filtration rates in children and young adults with chronic kidney disease. Kidney Int 99:948–956. 10.1016/j.kint.2020.10.047 33301749 10.1016/j.kint.2020.10.047 PMC9083470

[14] Kidney Disease: Improving Global Outcomes CKDWG (2024) KDIGO 2024 clinical practice guideline for the evaluation and management of chronic kidney disease. Kidney Int 105:S117–S314. 10.1016/j.kint.2023.10.018 38490803 10.1016/j.kint.2023.10.018

[15] Viteri B, Reid-Adam J (2018) Hematuria and proteinuria in children. Pediatr Rev 39:573–587. 10.1542/pir.2017-0300 30504250 10.1542/pir.2017-0300 PMC6494107

[16] Marsh MC, Junquera GY, Stonebrook E, Spencer JD, Watson JR (2024) Urinary tract infections in children. Pediatr Rev 45:260–270. 10.1542/pir.2023-006017 38689106 10.1542/pir.2023-006017

[17] Flynn JT, Kaelber DC, Baker-Smith CM et al (2017) Clinical practice guideline for screening and management of high blood pressure in children and adolescents. Pediatrics 140:1–72. 10.1542/peds.2017-1904 10.1542/peds.2017-1904 28827377

[18] Mina R, von Scheven E, Ardoin SP et al (2012) Consensus treatment plans for induction therapy of newly diagnosed proliferative lupus nephritis in juvenile systemic lupus erythematosus. Arthritis Care Res 64:375–383. 10.1002/acr.21558 10.1002/acr.21558 PMC3457803 22162255

[19] Harris PA, Taylor R, Thielke R, Payne J, Gonzalez N, Conde JG (2009) Research electronic data capture (REDCap) - a metadata-driven methodology and workflow process for providing translational research informatics support. J Biomed Inform 42:377–381. 10.1016/j.jbi.2008.08.010 18929686 10.1016/j.jbi.2008.08.010 PMC2700030

[20] Harris PA, Taylor R, Minor BL et al (2019) The REDCap consortium: building an international community of software platform partners. J Biomed Inform 95:103208–103218. 10.1016/j.jbi.2019.103208 31078660 10.1016/j.jbi.2019.103208 PMC7254481

[21] Chen HG, Chen JS, Chen YS, Yin CH, Chen HC, Chiou YH (2023) Comparison of mycophenolic acid with cyclophosphamide for the treatment of pediatric lupus nephritis: a retrospective study from a tertiary center hospital in Taiwan. J Microbiol Immunol Infect 56:1105–1113. 10.1016/j.jmii.2023.08.003 37586916 10.1016/j.jmii.2023.08.003

[22] Cui L, Jiao J, Zhang Y et al (2024) FCGR3A-V158F gene polymorphism: a potential predictor for rituximab dosing optimization in Chinese patients with neuromyelitis optica spectrum disorder. Mult Scler Relat Disord 86:105600. 10.1016/j.msard.2024.105600 38579568 10.1016/j.msard.2024.105600

[23] Lim SH, Vaughan AT, Ashton-Key M et al (2011) Fc gamma receptor IIb on target B cells promotes rituximab internalization and reduces clinical efficacy. Blood 118:2530–2540. 10.1182/blood-2011-01-330357 21768293 10.1182/blood-2011-01-330357

[24] Robinson JI, Md Yusof MY, Davies V et al (2022) Comprehensive genetic and functional analyses of Fc gamma receptors influence on response to rituximab therapy for autoimmunity. EBioMedicine 86:104343. 10.1016/j.ebiom.2022.104343 36371989 10.1016/j.ebiom.2022.104343 PMC9663864

[25] Basu B, Roy B, Babu BG (2017) Efficacy and safety of rituximab in comparison with common induction therapies in pediatric active lupus nephritis. Pediatr Nephrol 32:1013–1021. 10.1007/s00467-017-3583-x 28191596 10.1007/s00467-017-3583-x

[26] Hogan J, Godron A, Baudouin V et al (2018) Combination therapy of rituximab and mycophenolate mofetil in childhood lupus nephritis. Pediatr Nephrol 33:111–116. 10.1007/s00467-017-3767-4 28780657 10.1007/s00467-017-3767-4

[27] De Mutiis C, Wenderfer SE, Orjuela A et al (2022) Defining renal remission in an international cohort of 248 children and adolescents with lupus nephritis. Rheumatology 61:2563–2571. 10.1093/rheumatology/keab746 34626102 10.1093/rheumatology/keab746

[28] Davidson JE, Fu Q, Ji B et al (2018) Renal remission status and longterm renal survival in patients with lupus nephritis: a retrospective cohort analysis. J Rheumatol 45:671–677. 10.3899/jrheum.161554 29496892 10.3899/jrheum.161554 PMC5932209

[29] Chalhoub NE, Wenderfer SE, Levy DM et al (2022) International consensus for the dosing of corticosteroids in childhood-onset systemic lupus erythematosus with proliferative lupus nephritis. Arthritis Rheumatol 74:263–273. 10.1002/art.41930 34279063 10.1002/art.41930 PMC8766607

[30] Russo K (2022) Assessment and treatment of adolescents with chronic medical conditions. Journal of Health Service Psychology 48:69–78. 10.1007/s42843-022-00059-4 35496918 10.1007/s42843-022-00059-4 PMC9034877

[31] McGill G, Ambrose N (2018) The management of lupus in young people. Br J Gen Pract 68:96–97. 10.3399/bjgp18X694805 29371316 10.3399/bjgp18X694805 PMC5774960

[32] Rygg M, Pistorio A, Ravelli A et al (2012) A longitudinal PRINTO study on growth and puberty in juvenile systemic lupus erythematosus. Ann Rheum Dis 71:511–517. 10.1136/annrheumdis-2011-200106 21998114 10.1136/annrheumdis-2011-200106

[33] Mehta B, Jannat-Khah D, Glaser KK et al (2023) Fetal and maternal morbidity in pregnant patients with lupus: a 10-year US nationwide analysis. RMD Open 9:e002752. 10.1136/rmdopen-2022-002752 37185223 10.1136/rmdopen-2022-002752 PMC10255159

[34] Valenzuela-Almada MO, Hocaoglu M, Dabit JY et al (2022) Epidemiology of childhood-onset systemic lupus erythematosus: a population-based study. Arthritis Care Res 74:728–732. 10.1002/acr.24827 10.1002/acr.24827 PMC9038612 34825516

[35] Pereira M, Muscal E, Eldin K et al (2017) Clinical presentation and outcomes of childhood-onset membranous lupus nephritis. Pediatr Nephrol 32:2283–2291. 10.1007/s00467-017-3743-z 28717937 10.1007/s00467-017-3743-z

[36] Furie RA, Rovin BH, Houssiau F et al (2020) Two-year, randomized, controlled trial of belimumab in lupus nephritis. N Engl J Med 383:1117–1128. 10.1056/NEJMoa2001180 32937045 10.1056/NEJMoa2001180

[37] Rovin BH, Teng YKO, Ginzler EM et al (2021) Efficacy and safety of voclosporin versus placebo for lupus nephritis (AURORA 1): a double-blind, randomised, multicentre, placebo-controlled, phase 3 trial. Lancet 397:2070–2080. 10.1016/S0140-6736(21)00578-X 33971155 10.1016/S0140-6736(21)00578-X

[38] Saxena A, Ginzler EM, Gibson K et al (2024) Safety and efficacy of long-term voclosporin treatment for lupus nephritis in the Phase 3 AURORA 2 Clinical Trial. Arthritis Rheumatol 76:59–67. 10.1002/art.42657 37466424 10.1002/art.42657

[39] Furie RA, Rovin BH, Garg JP et al (2025) Efficacy and safety of obinutuzumab in active lupus nephritis. N Engl J Med 392:1471–1483. 10.1056/NEJMoa2410965 39927615 10.1056/NEJMoa2410965

