# Supplementary material for: Clinical presentations, treatments, and outcomes of pediatric lupus nephritis: a prospective cohort study from the Pediatric Nephrology Research Consortium
Source: Pediatr Nephrol. 2026 May 11;41(10):3543–57. doi: 10.1007/s00467-026-07329-z (PMC13481483; doi:10.1007/s00467-026-07329-z)
Supplement: Supplementary file 1 [file 467_2026_7329_MOESM2_ESM.docx]

Supplemental Online Content

Clinical Presentations, Treatments, and Outcomes of Pediatric Lupus Nephritis: A Prospective Cohort Study from the Pediatric Nephrology Research Consortium

Melonie Phillips, MD^1^; Mahmoud Kallash, MD^1^; Chloe Tang, MS, PhD^2^; Steve Rust, PhD^2^; Kia Jubert-Bacon, BS, MHA^3^; Scott Wenderfer, MD^3^; Smriti Mohan, MD^4^; Neal Blatt MD^4^; Don Batisky, MD^5^; Alejandro Quiroga, MD^6^; Tetyana L. Vasylyeva^7^, MD; Nilka DeJesus, MD^8^; Jerome Lane, MD^9^

Supplemental Table 1. Demographics and patient characteristics for the subset of 71 patients who received mycophenolate (MMF) or IV cyclophosphamide (CYC), both with or without Rituximab (RTX), as part of their initial therapy.

Supplemental Table 2. Laboratory test data, presence of any stage of acute kidney injury at time of diagnosis, presence of chronic kidney disease at ≥6 months of follow-up, presence of hypertension at time of diagnosis and at ≥6 months of follow-up for patients who received either mycophenolate (MMF) or IV cyclophosphamide (CYC), both with and without Rituximab (RTX), as part of their initial therapy.

Supplemental Table 3. Percentage of patients who achieved complete response (CR) and any response (CR or partial response [PR]) at 6-, 12-, and 24-month follow-up after receiving mycophenolate (MMF) with or without Rituximab (RTX) as part of their initial therapy.

Supplemental Table 4. Percentage of patients who achieved complete response (CR) and any response (CR or partial response [PR]) at 6-, 12-, and 24-month follow-up after receiving IV cyclophosphamide (CYC) with or without Rituximab (RTX) as part of their initial therapy.

Supplemental Table 5. Rates of corticosteroid use at 6-, 12-, and 24-month follow-up.

Supplemental Table 6. Rates of infections at 6-, 12-, and 24-month follow-up.

Supplemental Table 7. Rates of pediatric lupus nephritis flares at 6-, 12-, and 24-month follow-up.

Supplemental Table 1. Demographics and patient characteristics the subsets based on initial therapy received, including the 33 patients who received corticosteroids (CS) and mycophenolate (MMF) (labeled MMF); the 18 patients who received CS and IV cyclophosphamide (CYC) (labeled CYC); the 9 patients who received CS, MMF, and Rituximab (RTX) (labeled MMF+RTX); and the 11 patients cohort who received CS, CYC, and RTX (labeled CYC+RTX). For categorical values, number of affected patients and the corresponding percentages are provided. For numerical data, median values and their corresponding lower quartiles (Q1) and upper quartiles (Q3) are provided.

|  | **MMF** | **MMF+RTX** | **p-value^a^** | **CYC** | **CYC+RTX** | **p-value^b^** |
| --- | --- | --- | --- | --- | --- | --- |
| **Number of patients** | 33 | 9 |  | 18 | 11 |  |
| **Median age in years at SLE diagnosis (Q1, Q3)** | 14.5 (12, 16) | 15 (14, 17) |  | 13 (11, 16) | 12 (11, 15) |  |
| **Median age in years at pLN diagnosis (Q1, Q3)** | 15 (13,16) | 15 (14,17) | 0.40 | 14 (12,16) | 12 (11,15) | 0.36 |
| **Sex assigned at birth** |  |  | 0.59 |  |  | 0.62 |
| Female | 29 (88%) | 7 (78%) |  | 16 (89%) | 9 (82%) |  |
| Male | 4 (12%) | 2 (22%) |  | 2 (11%) | 2 (18%) |  |
| **Self-identified race** |  |  | 0.44 |  |  | 0.77 |
| African American | 12 (36%) | 3 (33%) |  | 5 (28%) | 3 (27%) |  |
| Asian^c^ | 3 (9%) | 0 (0%) |  | 3 (17%) | 0 (0%) |  |
| Caucasian | 9 (27%) | 1 (11%) |  | 3 (17%) | 2 (19%) |  |
| Hispanic | 5 (15%) | 4 (45%) |  | 4 (22%) | 3 (27%) |  |
| Other^d^ | 4 (12%) | 1 (11%) |  | 3 (17%) | 3 (27%) |  |
| **Insurance type** |  |  | 0.55 |  |  | 0.071 |
| Medicaid | 16 (49%) | 4 (45%) |  | 6 (33%) | 9 (82%) |  |
| Private | 12 (36%) | 4 (44%) |  | 11 (61%) | 2 (18%) |  |
| Self-pay | 1 (3%) | 1 (11%) |  | 0 (0%) | 0 (0%) |  |
| Not reported | 4 (12%) | 0 (0%) |  | 1 (6%) | 0 (0%) |  |
| **Biopsy class** |  |  | 0.28 |  |  | 0.37 |
| Class III | 7 (21%) | 4 (45%) |  | 2 (11%) | 3 (27%) |  |
| Class IV | 8 (24%) | 0 (0%) |  | 12 (67%) | 5 (46%) |  |
| Mixed class^e^ | 6 (18%) | 1 (11%) |  | 4 (22%) | 2 (18%) |  |
| Pure class V | 12 (36%) | 4 (44%) |  | 0 (0%) | 1 (9%) |  |

Abbreviations: CYC – IV cyclophosphamide; MMF – mycophenolate; pLN – pediatric lupus nephritis; RTX – rituximab; SLE – systemic lupus erythematosus ^a^These p-values are for comparisons between the MMF and MMF+RTX subsets.
^b^These p-values are for comparisons between the CYC and CYC+RTX subsets.
^c^Asian race includes East Asian and South Asian.
^d^Other constitutes either heterogeneous race (≥2 races were reported), or race was not reported.
^e^Mixed class denotes class V concomitant with either class III or IV.

Supplemental Table 2. Laboratory test data, presence of any stage of acute kidney injury (AKI) at time of diagnosis, presence of chronic kidney disease (CKD) at ≥6 months of follow-up, and presence of hypertension (HTN) at time of diagnosis and at ≥6 months of follow-up for patients who received: corticosteroids (CS) and mycophenolate (MMF) (labeled MMF); CS and IV cyclophosphamide (CYC) (labeled CYC); CS, MMF, and Rituximab (RTX) (labeled MMF+RTX); and CS, CYC, and RTX (labeled CYC+RTX). For categorical values, number of affected patients out of the total number of patients for which data were available and the corresponding percentages are provided. For numerical data, median values, their corresponding lower quartiles (Q1) and upper quartiles (Q3), and number of patients for which data were available are provided.

|  | **MMF (n=33)** | **MMF+RTX (n=9)** | **p-value^a^** | **CYC (n=18)** | **CYC+RTX (n=11)** | **p-value^b^** |
| --- | --- | --- | --- | --- | --- | --- |
| **Time of Diagnosis** | | | | | | |
| Hematuria present^c^ | 23/28 (82%) | 8/9  (89%) | 1.00 | 17/17 (100%) | 10/11  (91%) | 0.39 |
| Pyuria present^d^ | 7/28 (25%) | 4/9  (44%) | 0.40 | 8/17 (47%) | 10/11  (91%) | 0.041* |
| UPCR (mg/mg) |  |  | 0.45 |  |  | 0.69 |
| UPCR >0.2-≤2 | 15/33 (45%) | 6/9  (67%) |  | 7/18 (39%) | 3/11  (27%) |  |
| UPCR >2 | 18/33 (55%) | 3/9  (33%) |  | 11/18 (61%) | 8/11  (73%) |  |
| Albumin (g/dL) | 3.1 (2.5-3.5) n=32 | 2.6  (2.3-2.8) n=9 | 0.32 | 2.9 (2.2-3.2) n=18 | 2.1  (2.0-2.9) n=11 | 0.23 |
| C3 (mg/dL) | 81 (42-109) n=33 | 52  (33-65) n=9 | 0.082 | 42 (32-55) n=16 | 39  (26-67) n=11 | 0.74 |
| eGFR (mL/min/1.73m^2^) | 107 (91-123) n=33 | 91  (63-116) n=9 | 0.73 | 76 (62-99) n=18 | 58  (51-71) n=11 | 0.064 |
| AKI present^e^ | 6/33 (18%) | 3/9  (33%) | 0.37 | 11/18 (61%) | 10/11  (91%) | 0.10 |
| HTN present^f^ | 14/19 (42%) | 4/9  (44%) | 1.00 | 12/17 (71%) | 7/11  (64%) | 1.00 |
| **6-month follow-up** | | | | | | |
| Hematuria present^c^ | 11/21 (52%) | 4/8  (50%) | 1.00 | 10/15 (67%) | 7/10  (70%) | 1.00 |
| Pyuria present^d^ | 2/21 (10%) | 0/8  (0%) | 1.00 | 1/15 (7%) | 2/10  (20%) | 0.54 |
| UPCR (mg/mg) |  |  | 0.55 |  |  | 0.72 |
| UPCR ≤0.2 | 12/23 (52%) | 3/8  (38%) |  | 4/15 (27%) | 3/10  (30%) |  |
| UPCR >0.2-≤2 | 10/23 (44%) | 4/8  (50%) |  | 10/15 (66%) | 5/10  (50%) |  |
| UPCR >2 | 1/23 (4%) | 1/8  (12%) |  | 1/15 (7%) | 2/10  (20%) |  |
| Albumin (g/dL) | 3.9 (3.8-4.4) n=31 | 4.1  (3.8-4.3) n=9 | 0.83 | 4.0 (3.8-4.2) n=16 | 4.2  (4.1-4.5) n=11 | 0.14 |
| C3 (mg/dL) | 102 (88-132) n=29 | 127  (94-133) n=9 | 0.62 | 118 (81-128) n=16 | 117  (79-125) n=10 | 0.90 |
| eGFR (mL/min/1.73m^2^) | 107 (93-119) n=30 | 119  (100-154) n=9 | 0.41 | 100 (87-106) n=16 | 83  (79-109) n=11 | 0.43 |
| CKD present^g^ |  |  | 1.00 |  |  | 0.061 |
| Stage 1 | 24/29 (83%) | 7/9  (78%) |  | 12/16 (75%) | 4/11  (36%) |  |
| Stages 2-5 | 5/29 (17%) | 2/9  (22%) |  | 4/16 (25%) | 7/11  (64%) |  |
| HTN present^f^ | 8/31 (26%) | 2/9  (22%) | 1.00 | 4/16 (25%) | 2/11  (18%) | 1.00 |
| **12-month follow-up** | | | | | | |
| Hematuria present^c^ | 6/21 (29%) | 3/6  (33%) | 1.00 | 7/14 (50%) | 8/11  (73%) | 0.41 |
| Pyuria present^d^ | 2/21 (10%) | 2/9  (22%) | 0.56 | 2/14 (14%) | 0/11  (0%) | 0.49 |
| UPCR (mg/mg) |  |  | 0.36 |  |  | 0.30 |
| UPCR ≤0.2 | 13/22 (59%) | 3/7  (43%) |  | 10/14 (72%) | 6/10  (60%) |  |
| UPCR >0.2-≤2 | 6/22 (27%) | 4/7  (57%) |  | 2/14 (14%) | 4/10  (40%) |  |
| UPCR >2 | 3/22 (14%) | 0/7  (0%) |  | 2/14 (14%) | 0/10  (0%) |  |
| Albumin (g/dL) | 4.1 (4.0-4.6) n=23 | 4.0  (4.0-4.4) n=9 | 0.53 | 4.4 (4.1-4.4) n=16 | 4.4  (4.1-4.6) n=10 | 0.52 |
| C3 (mg/dL) | 105 (86-133) n=25 | 128  (101-134) n=9 | 0.20 | 108 (85-126) n=15 | 116  (104-129) n=10 | 0.25 |
| eGFR (mL/min/1.73m^2^) | 104 (96-126) n=27 | 97  (82-116) n=9 | 0.28 | 102 (92-121) n=17 | 88  (71-111) n=10 | 0.16 |
| CKD present^g^ |  |  | 0.58 |  |  | 0.38 |
| Stage 1 | 24/27 (89%) | 7  (78%) |  | 13/17 (76%) | 5/9  (56%) |  |
| Stages 2-5 | 3/27 (11%) | 2  (22%) |  | 4/17 (24%) | 4/9  (44%) |  |
| HTN present^f^ | 3/26 (12%) | 3/9  (33%) | 0.16 | 2/17 (12%) | 3/11  (27%) | 0.35 |
| **24-month follow-up** | | | | | | |
| Hematuria present^c^ | 5/18 (28%) | 5/7  (71%) | 0.075 | 3/8 (38%) | 3/8  (38%) | 1.00 |
| Pyuria present^d^ | 3/18 (17%) | 2/7  (29%) | 0.60 | 1/8 (13%) | 1/8  (13%) | 1.00 |
| UPCR (mg/mg) |  |  | 0.21 |  |  | 0.80 |
| UPCR ≤0.2 | 8/14 (57%) | 6/7  (86%) |  | 4/8 (50%) | 5/10  (50%) |  |
| UPCR >0.2-≤2 | 1/14 (7%) | 1/7  (14%) |  | 3/8 (38%) | 5/10  (50%) |  |
| UPCR >2 | 5/14 (36%) | 0/7  (0%) |  | 1/8 (12%) | 0/10  (0%) |  |
| Albumin (g/dL) | 4.1 (4.0-4.4) n=17 | 4.2  (3.9-4.5) n=7 | 1.00 | 4.5 (4.3-4.8) n=9 | 4.5  (4.3-4.6) n=9 | 0.50 |
| C3 (mg/dL) | 105 (91-128) n=21 | 107  (71-130) n=7 | 0.72 | 110 (80-124) n=9 | 119  (117-125) n=9 | 0.30 |
| eGFR (mL/min/1.73m^2^) | 103 (94-127) n=21 | 138  (115-152) n=7 | 0.032 | 105 (98-121) n=9 | 102  (93-110) n=9 | 0.40 |
| CKD present^g^ |  |  | 0.55 |  |  | 1.00 |
| Stage 1 | 17/21 (81%) | 7/7 (100%) |  | 8/9 (89%) | 7/9  (78%) |  |
| Stages 2-5 | 4/21 (19%) | 0/7  (0%) |  | 1/9 (11%) | 2/9  (22%) |  |
| HTN present^f^ | 6/21 (29%) | 0/7  (0%) | 0.29 | 0/7 (100%) | 1/9  (11%) | 1.00 |

Abbreviations: AKI – acute kidney injury; CKD – chronic kidney disease; CYC – IV cyclophosphamide; eGFR – estimated glomerular function rate; HTN – hypertension; MMF – mycophenolate; UPCR – urine protein-to-creatinine ratio
^a^These p-values are for comparisons between the MMF and MMF+RTX subsets.
^b^These p-values are for comparisons between the CYC and CYC+RTX subsets.
^c^Hematuria was defined as urine RBC ≥3/high powered field.
^d^Pyuria was defined as urine WBC ≥5/ high powered field.
^e^AKI staging definitions used are from the KDIGO 2012 AKI guidelines.^11^
^f^HTN was defined as either systolic blood pressure (SBP) or diastolic blood pressure (DBP) ≥95th percentile for patients aged <13 years and SBP ≥130 or DBP ≥80 for patients aged ≥13 years.^17
g^CKD staging definitions used are from the KDIGO 2024 CKD guidelines.^14^

Supplemental Table 3. Percentage of patients who achieved complete response (CR) and any response (CR or partial response [PR]) at 6-, 12-, and 24-month follow-ups after receiving initial therapy with corticosteroids (CS) and mycophenolate (MMF) with or without Rituximab (RTX) regardless of pediatric lupus nephritis (pLN) biopsy class (labeled as “all”) and stratified by pLN biopsy class. Number of patients who achieved the specified response out of the total number of patients for which data were available and the corresponding percentages are provided. Dashed lines denote that there were no available data for that time point. P-values are listed as not applicable (N/A) when data were not available for comparison or the comparison was between data with identical values.

|  |  | **Complete Response** | | | **Any Response (CR or PR)** | | |
| --- | --- | --- | --- | --- | --- | --- | --- |
| **pLN class** | **Initial therapy** | 6 months | 12 months | 24 months | 6 months | 12 months | 24 months |
| **All** | MMF | 11/24 (46%) | 13/22 (59%) | 7/14 (50%) | 21/24 (88%) | 18/22 (82%) | 7/14 (50%) |
|  | MMF+RTX | 3/8 (38%) | 2/7 (29%) | 6/7 (86%) | 6/8 (75%) | 3/7 (43%) | 7/7 (100%) |
|  | p-value | 1.00 | 0.22 | 0.17 | 0.58 | 0.068 | 0.047* |
| **III** | MMF | 2/5 (40%) | 5/5 (100%) | 3/4 (75%) | 3/5 (60%) | 5/5 (100%) | 3/4 (75%) |
|  | MMF+RTX | 3/4 (75%) | 1/3 (33%) | 3/3 (100%) | 3/4 (75%) | 2/3 (67%) | 3/3 (100%) |
|  | p-value | 0.52 | 0.11 | 1.00 | 1.00 | 0.38 | 1.00 |
| **IV** | MMF | 2/5 (40%) | 2/4 (50%) | 1/3 (33%) | 5/5 (100%) | 3/4 (75%) | 1/3 (33%) |
|  | MMF+RTX^a^ | - | - | - | - | - | - |
|  | p-value | N/A | N/A | N/A | N/A | N/A | N/A |
| **Mixed class^b^** | MMF | 3/4 (75%) | 3/5 (60%) | 1/2 (50%) | 4/4 (100%) | 4/5 (80%) | 1/2 (50%) |
|  | MMF+RTX | 0/1 (0%) | - | 0/1 (0%) | 1/1 (100%) | - | 1/1 (100%) |
|  | p-value | 0.40 | N/A | 1.00 | N/A | N/A | 1.00 |
| **Pure class V** | MMF | 3/9 (33%) | 3/8 (38%) | 2/5 (40%) | 8/9 (89%) | 6/8 (75%) | 2/5 (40%) |
|  | MMF+RTX | 0/3 (0%) | 1/4 (25%) | 3/3 (100%) | 2/3 (67%) | 1/4  (25%) | 3/3 (100%) |
|  | p-value | 0.50 | 1.00 | 0.20 | 0.42 | 0.22 | 0.20 |

Abbreviations: CR – complete response; MMF – mycophenolate; PR – partial response; pLN – pediatric lupus nephritis; N/A – not applicable; RTX – rituximab
^a^No patients with class IV pLN received MMF with RTX.
^b^Mixed class denotes class V concomitant with either class III or IV.

Supplemental Table 4. Percentage of patients who achieved complete response (CR) and any response (CR or partial response [PR]) at 6-, 12-, and 24-month follow-ups after receiving initial therapy with corticosteroids (CS) and IV cyclophosphamide (CYC) with or without Rituximab (RTX) regardless of pediatric lupus nephritis (pLN) biopsy class (labeled as “all”) and stratified by pLN biopsy class. Number of patients who achieved the specified response out of the total number of patients for which data were available and the corresponding percentages are provided. Dashed lines denote that there were no available data for that time point. P-values are listed as not applicable (N/A) when data were not available for comparison or the comparison was between data with identical values.

|  |  | **Complete Response** | | | **Any Response (CR or PR)** | | |
| --- | --- | --- | --- | --- | --- | --- | --- |
| **pLN class** | **Initial therapy** | 6 months | 12 months | 24 months | 6 months | 12 months | 24 months |
| **All** | CYC | 4/15 (27%) | 10/14 (71%) | 4/8 (50%) | 13/15 (87%) | 12/14 (86%) | 7/8 (88%) |
|  | CYC+RTX | 2/10 (20%) | 4/9 (44%) | 5/9 (56%) | 7/10 (70%) | 8/9 (89%) | 9/9 (100%) |
|  | p-value | 1.00 | 0.21 | 1.00 | 0.36 | 1.00 | 0.47 |
| **III** | CYC | 1/1 (100%) | 2/2 (100%) | - | 1/1 (100%) | 2/2 (100%) | - |
|  | CYC+RTX | 1/3 (33%) | 2/3 (67%) | 1/3 (33%) | 3/3 (100%) | 3/3 (100%) | 3/3 (100%) |
|  | p-value | 1.00 | 1.00 | N/A | 1.00 | 1.00 | N/A |
| **IV** | CYC | 3/11 (27%) | 7/10 (70%) | 4/6 (67%) | 10/11 (91%) | 8/10 (80%) | 6/6 (100%) |
|  | CYC+RTX | 1/5 (20%) | 0/4 (0%) | 3/4 (75%) | 3/5 (60%) | 3/4 (75%) | 4/4 (100%) |
|  | p-value | 1.00 | 0.026* | 1.00 | 1.00 | 1.00 | 1.00 |
| **Mixed class^a^** | CYC | 0/3 (0%) | 1/2 (50%) | 0/2 (0%) | 2/3 (67%) | 2/2 (100%) | 1/2 (50%) |
|  | CYC+RTX | 0/2 (0%) | 2/2 (100%) | 1/2 (50%) | 1/2 (50%) | 2/2 (100%) | 2/2 (100%) |
|  | p-value | N/A | 1.00 | 1.00 | 1.00 | 1.00 | 1.00 |
| **Pure class V** | CYC^b^ | - | - | - | - | - | - |
|  | CYC+RTX^c^ | - | - | - | - | - | - |
|  | p-value | N/A | N/A | N/A | N/A | N/A | N/A |

Abbreviations: CYC – IV cyclophosphamide; pLN – pediatric lupus nephritis; N/A – not applicable; RTX – rituximab
^a^Mixed class denotes class V concomitant with either class III or IV.

^b^No patients with pure class V pLN received CYC.
^c^One patient with pure class V pLN received CYC with RTX but did not have laboratory values documented at follow-up.

Supplemental Table 5. Rates of corticosteroid (CS) use at 6-, 12-, and 24-month follow-up for patients **A.** regardless of initial therapy received; **B.** after receiving either mycophenolate (MMF) or IV cyclophosphamide (CYC) for initial therapy; and **C.** after receiving Rituximab (RTX) or no RTX along with either MMF or CYC for initial therapy. Number of affected patients out of the total number of patients for which data were available and the corresponding percentages are provided.

|  | **A** | **B** | | | **C** | | |
| --- | --- | --- | --- | --- | --- | --- | --- |
| **Time of follow-up** | **All patients** | **MMF** | **CYC** | **p-value^a^** | **No RTX** | **RTX** | **p-value^b^** |
| 6 months | 62/67 (93%) | 26/31 (84%) | 16/16 (100%) | 0.15 | 42/47 (89%) | 20/20 (100%) | 0.31 |
| 12 months | 51/64 (80%) | 19/27 (70%) | 15/17 (88%) | 0.27 | 34/44 (77%) | 17/20 (85%) | 0.74 |
| 24 months | 24/47 (51%) | 11/21 (52%) | 6/9 (67%) | 0.69 | 17/30 (57%) | 7/17 (41%) | 0.37 |

Abbreviations: CYC – IV cyclophosphamide; MMF – mycophenolate; RTX – rituximab
^a^These p-values are for the comparisons between MMF and CYC subsets.
^b^These p-values are for the comparisons between no RTX and RTX subsets.

Supplemental Table 6. Rates of infections at 6-, 12-, and 24-month follow-up for patients **A.** regardless of initial therapy received; **B.** after receiving either mycophenolate (MMF) or IV cyclophosphamide (CYC) for initial therapy; and **C.** after receiving Rituximab (RTX) or no RTX along with either MMF or CYC for initial therapy. Number of affected patients out of the total number of patients for which data were available and the corresponding percentages are provided.

|  | **A** | **B** | | | **C** | | |
| --- | --- | --- | --- | --- | --- | --- | --- |
| **Time of follow-up** | **All patients** | **MMF** | **CYC** | **p-value^a^** | **No RTX** | **RTX** | **p-value^b^** |
| 6 months | 9/67 (13%) | 3/31 (10%) | 1/16 (6%) | 1.00 | 4/47 (9%) | 5/20 (25%) | 0.11 |
| 12 months | 8/64 (13%) | 2/27 (7%) | 1/17 (6%) | 1.00 | 3/44 (7%) | 5/20 (25%) | 0.096 |
| 24 months | 10/47 (21%) | 5/21 (24%) | 1/9 (11%) | 0.64 | 6/30 (20%) | 4/17 (24%) | 1.00 |

Abbreviations: CYC – IV cyclophosphamide; MMF – mycophenolate; RTX – rituximab
^a^These p-values are for the comparisons between MMF and CYC subsets.
^b^These p-values are for the comparisons between no RTX and RTX subsets.

Supplemental Table 7. Rates of pediatric lupus nephritis (pLN) flares at 6-, 12-, and 24-month follow-up for patients **A.** regardless of initial therapy received; **B.** after receiving either mycophenolate (MMF) or IV cyclophosphamide (CYC) for initial therapy; and **C.** after receiving Rituximab (RTX) or no RTX along with either MMF or CYC for initial therapy. Number of affected patients out of the total number of patients for which data were available and the corresponding percentages are provided.

|  | **A** | **B** | | | **C** | | |
| --- | --- | --- | --- | --- | --- | --- | --- |
| **Time of follow-up** | **All patients** | **MMF** | **CYC** | **p-value^a^** | **No RTX** | **RTX** | **p-value^b^** |
| 6 months | 18/67 (27%) | 5/31 (16%) | 3/16 (19%) | 1.00 | 8/47 (17%) | 10/20 (50%) | 0.014* |
| 12 months | 11/64 (17%) | 4/27 (15%) | 3/17 (17%) | 1.00 | 7/44 (16%) | 4/20 (20%) | 0.73 |
| 24 months | 6/47 (13%) | 1/21 (5%) | 2/9 (22%) | 0.21 | 3/30 (10%) | 3/17 (18%) | 0.65 |

Abbreviations: CYC – IV cyclophosphamide; MMF – mycophenolate; RTX – rituximab
^a^These p-values are for the comparisons between MMF and CYC subsets.
^b^These p-values are for the comparisons between no RTX and RTX subsets.
